# Acute Pancreatitis Induced by Linagliptin: A Rare but Dangerous Side Effect

**DOI:** 10.7759/cureus.14104

**Published:** 2021-03-25

**Authors:** Artem Sharko, Shirly Samuel, Nikita Jain

**Affiliations:** 1 Internal Medicine, Northwestern Medicine McHenry Hospital, McHenry, USA

**Keywords:** dpp-4 inhibitors, pancreatitis, diabetes, linagliptin, side effect

## Abstract

Diabetes mellitus type 2 (DMT2) is a highly prevalent disease both in the United States and worldwide. Multiple treatment options are currently available, and several new groups of medications have been introduced over the last couple of decades. One of these groups is dipeptidyl peptidase-4 (DPP-4) inhibitors. These medications have side effects, some of which are severe and potentially life-threatening; however, some of their side effects have been underreported since they are relatively new. When prescribing these medications, it is essential to be cautious, especially with patients at an increased risk of developing an adverse effect. We present the case of a 57-year-old male who developed DPP-4 inhibitor-induced acute pancreatitis after the initiation of linagliptin. The patient did not have any apparent risk factors for pancreatitis as he did not drink alcohol or smoke cigarettes, his lipid panel was within normal limits, and he had a cholecystectomy five years prior. His linagliptin was held in the hospital and discontinued post-discharge, which led to the resolution of his symptoms.

## Introduction

Dipeptidyl peptidase-4 (DPP-4) inhibitors, also called gliptins, are incretin mimetics that activate glucagon-like peptide-1 (GLP-1) receptors and increase glucose-dependent insulin release by pancreatic beta cells [1]. FDA-approved DPP-4 inhibitors include linagliptin, sitagliptin, saxagliptin, and alogliptin [2]. They are widely used for the treatment of diabetes mellitus type 2 (DMT2) as monotherapy or combined with other antidiabetic medications. A rare yet potentially severe adverse effect of this group of medications is the development of acute pancreatitis.

## Case presentation

**Figure 1:**
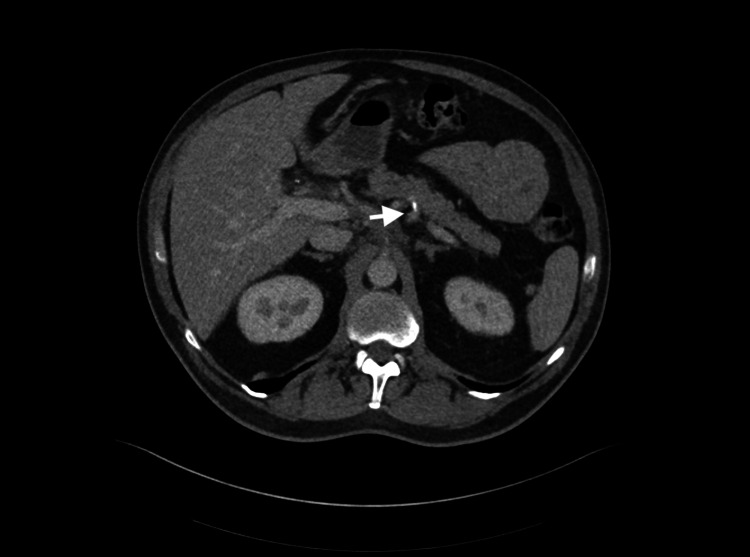
CT of the abdomen: mild haziness within the peripancreatic fat (white arrow) extending into the mesenteric root.

A 57-year-old male with a past medical history of DMT2 managed with metformin, linagliptin, and insulin glargine presented with three weeks of progressively worsening epigastric pain. The pain was constant, 10/10 in intensity, and was radiating to the back. He denied vomiting, diarrhea, or constipation; however, he reported nausea and floating, oily stools. The patient used over-the-counter nonsteroidal anti-inflammatory drugs with no effect. His past medical history was also remarkable for gallstone-induced pancreatitis managed with cholecystectomy five years prior. Social history was negative for alcohol use, cigarette smoking, or recreational drug use. The only recent change was the addition of linagliptin to his antidiabetic regimen approximately three months prior. On presentation, his blood pressure was elevated, but otherwise, vital signs were within normal limits. The physical examination was significant for severe tenderness to palpation in the epigastrium with guarding. Routine laboratory investigations, including complete blood count, basic metabolic panel, and liver function test, were unremarkable. Lipase was elevated at 1059 U/L. Lipid panel showed triglycerides 166 mg/dL, LDL 49 mg/dL, HDL 34 mg/dL and total cholesterol 116 mg/dL. Ranson's score was two on admission and remained the same at 48 hours. CT of the abdomen was pertinent for haziness within the peripancreatic fat, extending into the mesenteric root (Figure 1). These findings were indicative of acute interstitial edematous pancreatitis.

**Figure 2:**
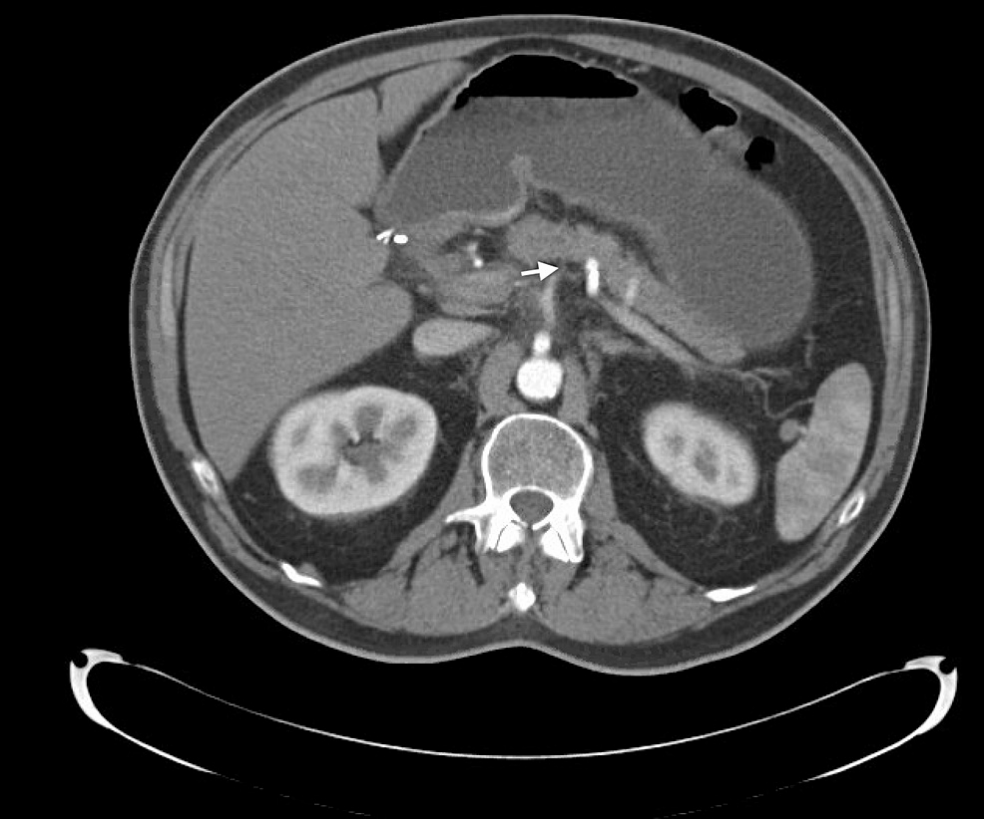
CT of the abdomen: interval resolution of previously seen fat stranding surrounding the pancreas (white arrow).

The patient received supportive care with bowel rest, opioid analgesics, and intravenous fluids. His antidiabetic medications were discontinued, and his diabetes was managed only with sliding scale insulin. The patient continued to have abdominal pain and tenderness on days two and three of hospitalization and required significant doses of opioids. His lipase trended down to 591 U/L on day two. Other laboratory investigations, including white blood cell count and liver function panel, remained unremarkable. On day four, the patient's pain improved significantly, and he could take a liquid diet. His diet was gradually advanced, and on day five of hospitalization, the patient was stable for discharge. On discharge, the patient's linagliptin was discontinued, but his metformin and insulin glargine were restarted. He was instructed to undergo repeat imaging of the pancreas and follow up with his endocrinologist to further manage diabetes in two weeks. The patient remained off of the linagliptin and had a repeat CT of the abdomen, which showed resolution of the stranding surrounding the pancreas with no peripancreatic fluid collection (Figure 2).

## Discussion

For a while, it was unclear whether DPP-4 inhibitors can cause acute pancreatitis, and the data from early studies were inconclusive [3]. However, by now, DPP-4 inhibitors have been used for over a decade, and numerous clinical trials have investigated their effects. A recent meta-analysis showed a 79% relative increase in acute pancreatitis incidence in patients using DPP-4 inhibitors [4]. The increase in absolute risk was 0.13%, indicating an increase in acute pancreatitis incidence by 1-2 cases for every 1000 patients on DPP-4 inhibitors [4]. A proposed mechanism of pancreatic injury by DPP-4 inhibitors is the increase of the half-life of GLP-1, which causes ductal hyperplasia in the pancreas with subsequent outflow obstruction and intrapancreatic activation of enzymes, leading to a state of chronic inflammation [5]. To the best of our knowledge, there have been only two case reports of linagliptin-induced pancreatitis so far and several reports for other DPP-4 inhibitors [6,7]. We believe that it is essential for clinicians to remember the possibility of developing acute pancreatitis in patients using DPP-4 inhibitors. It is also necessary to warn the patients of this risk and educate them on recognizing warning symptoms. Furthermore, data shows that recurrence of acute pancreatitis can be seen in 20-30% of patients, and the same etiologic factor does not necessarily cause the subsequent episode. It appears that in our case, the prior episode was a risk factor for developing another episode while on a DPP-4 inhibitor [8].

## Conclusions

DPP-4 inhibitors are popular drugs because of their excellent results in lowering blood glucose levels in diabetic patients, low risk of weight gain or hypoglycemia, and administration ease. However, these benefits do not come without some rare adverse effects. While acute pancreatitis in patients taking DPP-4 inhibitors is not commonly reported, in a patient who had a prior episode of acute pancreatitis, such as in our case, it is likely advisable to avoid prescribing a DPP-4 inhibitor or at least closely monitor for warning signs.
